# Study on key markers of NETosis: predictive indicator for postoperative frailty following complete cytoreductive surgery in advanced ovarian cancer

**DOI:** 10.3389/fimmu.2026.1769726

**Published:** 2026-04-16

**Authors:** Xianmei Lu, Feng Xu

**Affiliations:** Medical Center of Diagnosis and Treatment for Cervical Diseases, Wuxi Maternal and Child Health Hospital, Wuxi School of Medicine, Jiangnan University, Jiangsu, China

**Keywords:** advanced ovarian cancer, complete cytoreduction, frailty, netosis, prediction model

## Abstract

**Objective:**

This study analyzed factors associated with complete cytoreductive surgery and postoperative frailty in advanced ovarian cancer using key neutrophil extracellular trap (NETosis) markers—neutrophil elastase (NE), myeloperoxidase (MPO), and citrullinated histone H3 (Cit-H3). A risk prediction model was developed and validated.

**Methods:**

In this prospective cohort study, 189 advanced ovarian cancer patients (2020–2023) were classified into frail (n=41) and non-frail (n=148) groups based on postoperative status, and all patients were followed up for 2 years. Clinical data were collected, and risk factors for postoperative frailty in advanced ovarian cancer were identified using a machine learning method *(LASSO - XGBoost*). A nomogram−based prediction model was constructed. Internal validation and decision curve analysis confirmed favorable predictive efficacy and clinical net benefit of the model.

**Results:**

Significant differences were found between groups in age, education, marital status, daily activity, nutrition score, State-Trait Anxiety Inventory (STAI), Pittsburgh Sleep Quality Index (PSQI), NE, MPO, and Cit-H3 (P<0. 05). *Kaplan-Meier* survival curves showed that postoperative frailty was associated with worse prognosis (P<0. 05). Eight common risk factors were identified through overlapping screening by two machine learning methods, *LASSO* regression and *XGBoost*. Multivariate *Logisti*c regression confirmed age, STAI, MPO, NE, and Cit-H3 as independent risk factors (all *OR*>1, P<0. 05), while nutrition score was protective (OR<1, P<0. 05). The constructed nomogram model exhibited good discriminative ability (AUC = 0. 882) and calibration (C-index=0. 856, calibration slope=0. 92). The *Hosmer-Lemeshow* test indicated good model fit (P = 0. 893), and decision curve analysis demonstrated high net clinical benefit.

**Conclusion:**

Postoperative frailty in advanced ovarian cancer is associated with a multifactorial profile, primarily driven by age, nutritional status, STAI scores, MPO, NE, and Cit-H3 levels. The nomogram model was constructed based on these factors initially demonstrated favorable predictive efficacy, and it is expected to serve as an auxiliary tool for the early clinical identification of high-risk populations. However, this study is an exploratory single-center, small-sample research, and its conclusions still need to be further verified through external validation studies with multicenter and large-sample designs.

## Introduction

Ovarian cancer ranks as the most lethal gynecological malignancy. Primary ovarian neoplasms account for approximately 90% to 95% of cases, while the remaining 5% to 10% are attributed to metastases from extra-ovarian origins. Within this spectrum, high-grade serous carcinoma constitutes the most prevalent histological subtype (1). Fagotti et al. (2) reported that ovarian cancer is typically asymptomatic during its early phases. Symptoms resembling indigestion, such as dull lower abdominal pain, abdominal distension, and loss of appetite, generally manifest only upon progression to advanced stages. Consequently, the limited utility of current screening methods renders early diagnosis challenging, which results in 60–70 percent of patients presenting with advanced-stage disease when they seek medical advice. To tackle this crucial issue, the current standard approach to treatment primarily involves performing an initial surgery to achieve the greatest possible reduction of tumor cells. This surgical intervention is then followed by a postoperative combination chemotherapy regimen that utilizes platinum-based drugs. Achieving complete cytoreduction, defined as the absence of macroscopic residual disease, constitutes the strongest independent prognostic factor determining both progression-free survival and overall survival in patients with advanced ovarian cancer (3).

However, complete cytoreductive surgery typically entails extensive, multi-organ resection. The substantial trauma associated with this procedure poses a severe threat to patients’ physiological reserves, and postoperative frailty has consequently emerged as a prominent clinical concern. Moreno-Carmona et al. (4) defined postoperative frailty as a clinical state characterized by diminished physiological reserve and multi-system dysfunction. This condition results in heightened vulnerability to stressors and an increased risk of adverse outcomes. Pertinent research indicates that those with ovarian cancer who have undergone comprehensive cytoreductive surgery often display significant tiredness, muscle loss, poor nutrition, rapid decreases in physical mobility, and changes in cognitive function. These manifestations not only significantly compromise short-term recovery quality but are also closely correlated with increased rates of postoperative complications and readmission, poor chemotherapy tolerance, treatment delays or interruptions, tumor recurrence, and reduced overall survival (5). Therefore, the precise identification of patients at high risk for postoperative frailty, coupled with the implementation of preoperative interventions, is of pivotal significance for optimizing therapeutic strategies and improving long-term prognoses. The formation of neutrophil extracellular traps (NETosis) has been established as one of the critical pathological processes linking cancer, chronic inflammation and systemic functional decline. Key indicators of NETosis, including neutrophil elastase (NE), myeloperoxidase (MPO) and citrullinated histone H3 (Cit-H3), act as potent pro-inflammatory mediators. These molecules facilitate the generation of an immunosuppressive microenvironment and directly induce tissue injury and catabolism of muscle proteins. All of the above processes are highly consistent with the pathophysiological characteristics of frailty (6). Investigations indicate that key indicators of NETosis facilitate metastasis by physically entrapping cancer cells, promote tumor-associated thrombosis by activating coagulation pathways, and attenuate anti-tumor immunity by generating an immunosuppressive microenvironment. Furthermore, NETs and their components function as potent pro-inflammatory mediators that sustain inflammasome activation. This particular activity triggers a sequential discharge of pro - inflammatory cytokines and brings about a condition of widespread inflammation throughout the body (7). Accordingly, a hypothesis is proposed that plasma biomarker levels indicative of NETosis activity may be associated with the risk of frailty in patients with advanced ovarian cancer following major surgical trauma. Studies investigating the utility of circulating NETosis−related markers as a tool for predicting postoperative frailty remain scarce, particularly within the specific population of patients with advanced ovarian cancer.

Methodological innovations are clearly identified in this study. Analyses were conducted in patients with advanced ovarian cancer to address the limitations of traditional univariate screening. Two machine learning algorithms *LASSO* regression and extreme gradient boosting (*XGBoost*) were applied simultaneously in parallel to perform overlapping screening of clinical characteristics, psychological and nutritional indicators, and key NETosis markers. Multicollinearity was effectively addressed, and the robustness of the included variables was ensured. On this basis, variables identified by both screening methods were incorporated into multivariate *Logistic* regression analysis. A multidimensional risk prediction nomogram integrating psychological and nutritional indicators, key NETosis markers, and clinical characteristics was constructed for the first time. This nomogram aims to provide a novel quantitative tool and theoretical basis for the early identification and targeted intervention of postoperative frailty in patients with advanced ovarian cancer.

## Methods

### Study participants

This study was designed as a single-center, prospective observational cohort study aimed at investigating risk factors for frailty following complete cytoreductive surgery in patients with advanced ovarian cancer and establishing a predictive model. Study reporting was conducted in strict accordance with “the Strengthening the Reporting of Observational Studies in Epidemiology” (STROBE) statement. Patient enrollment was performed from January 1, 2020, to January 1, 2023. Written informed consent was obtained from all patients upon hospital admission, and baseline clinical data, laboratory parameters, and follow-up data were collected prospectively. The primary exposures were preoperative key markers of NETosis and psychosocial factors. The primary outcome was frailty status assessed within 6 months postoperatively according to the Tilburg Frailty Index (TFI). The sample size was determined using the formula n_c_=(*μ*_1-α_/2+*μ*_1-β_)^2^S^2^(1 + 1/*k*)/(*μ*_t_-*μ*_c_)_2_. Participants satisfying the inclusion and exclusion criteria were selected, and a total of 189 patients admitted between January 2020 and January 2023 were enrolled for prospective analysis. Based on their post - operative frailty condition, these patients were split into two groups: the frailty group, which consisted of 41 patients, and the non - frailty group, with 148 patients. All the recruited patients underwent treatment at the hospital. The research project obtained approval from the hospital’s Ethics Committee and was carried out in strict compliance with national laws and international standards.

### Inclusion, exclusion, exclusion/withdrawal, and early termination criteria

Inclusion Criteria: ①Individuals must be over 18 years of age; ②Patients should have pathologically verified primary epithelial ovarian malignant tumors that comply with the corresponding criteria outlined in the Clinical Application Guidelines for Molecular Pathological Testing of Ovarian Cancer (8); ③Histological categorizations include high - grade serous cancer, low - grade serous cancer, endometrioid cancer, clear cell cancer, mucinous cancer, carcinosarcoma, borderline neoplasms, and also mixed epithelial cancers. ④Completion of surgical resection; ⑤No severe neurological diseases or cognitive impairments.

Exclusion Criteria: ①Secondary ovarian carcinoma; ②Concurrent other malignant tumors; ③Palliative surgery performed for the correction of malignant intestinal obstruction, intestinal fistula, or other conditions; ④Previous history of malignant tumors; ⑤Severe dysfunction of vital organs such as the liver and kidneys. ⑦ preoperative active infection, recent major trauma or a history of recent major surgery. ⑥ preoperative frailty (TFI score ≥ 5).

Exclusion/withdrawal criteria: ①Unqualified sample quality; ②Missing follow-up data; ③Diagnosis of severe infections, end-stage renal disease, or other conditions that compromise the independence of prognostic assessment.

Early termination/withdrawal criteria from the study: ①Request for termination by the Ethics Committee or regulatory authorities; ②Identification of systematic operational errors in the study protocol that undermine the reliability of overall data; ③Withdrawal of informed consent by patients; ④Occurrence of severe allergic reactions, infections, or other unacceptable risks directly related to testing procedures or other study-related operations during the research.

### Treatment methods

All patients received standardized platinum−based comprehensive therapy. ① Surgical Procedures: Patients were placed in a supine frog-leg position, and general anesthesia was administered. A midline abdominal incision extending around the umbilicus was made, with the lower end reaching above the pubis and the upper end extending to below the xiphoid process. Comprehensive exploration was performed on the peritoneal surface of the diaphragmatic upper abdomen, liver surface, stomach, greater omentum, spleen, jejunum, ileum, appendix, colorectum, retroperitoneal lymph nodes, uterus, and bilateral adnexa. In cases of patients presenting indications for fertility conservation, an immediate assessment of the opposite adnexa was conducted. Subsequently, Total hysterectomy, bilateral adnexectomy, and greater omentectomy were implemented. Multivisceral resection of pelvic and abdominal implanted lesions, peritoneum, enlarged retroperitoneal lymph nodes, and even intestinal segments was performed when necessary. Following these procedures, peritoneal lavage was conducted using either normal saline or a 10% glucose solution to comprehensively cleanse the abdominal cavity of residual blood and tumor cells. Finally, hemostasis by suture, drainage tube placement, incision closure, and dressing application were completed sequentially. ② Chemotherapy regimens consisted of carboplatin at AUC of 5–6 mg/mL·min plus paclitaxel at 135–175 mg/m², or cisplatin at 75 mg/m² plus paclitaxel at 135–175 mg/m². Each treatment cycle was administered over a 21-day interval. Chemotherapy Procedures were as follows: Prior to chemotherapy, blood routine tests, biochemistry, electrocardiography, and other examinations were performed to assess the patient’s general condition and organ function. Antihistamines and antiemetic drugs were administered to prevent chemotherapy-related toxicities. The specific chemotherapy regimen and dosage were determined, with the required drug amount calculated based on the patient’s height, weight, and body surface area. During chemotherapy, drugs were delivered via intravenous infusion. Platinum-based agents were administered first, followed by paclitaxel-based drugs, with each infusion lasting 3–4 hours. After chemotherapy, blood routine tests and biochemistry were repeated to monitor treatment efficacy and toxicities. Fluid replacement therapy was provided to improve the patient’s nutritional status and immune function. Patients were assigned to one of two treatment pathways based on preoperative multidisciplinary team evaluation of tumor burden, resectability and general condition. A. Primary cytoreductive surgery: Maximal cytoreductive surgery was performed initially, followed by 6–8 cycles of adjuvant chemotherapy. B. Neoadjuvant chemotherapy followed by interval cytoreductive surgery: 3–4 cycles of neoadjuvant chemotherapy were administered following diagnosis, after which interval cytoreductive surgery was performed, with an additional 2–4 cycles of chemotherapy postoperatively. Surgical procedures were performed by experienced gynecologic oncologists with the goal of achieving complete cytoreduction. Surgical completeness was evaluated using the R classification system: R0 indicated no visible residual lesions; R1 indicated any visible residual lesions with a maximum diameter ≤ 1 cm; and R2 indicated any residual lesions with a maximum diameter > 1 cm. Complete cytoreductive surgery, as defined in this study, corresponded to R0 resection. All surgical records and postoperative imaging reports were independently reviewed by two senior physicians to verify residual disease status, with any disagreements resolved by a third specialist. Postoperative pathological staging was performed in accordance with the FIGO 2014 criteria.

### Basis for grouping

The evaluation was carried out using the Tilburg Frailty Indicator (TFI), initially devised by Gobbens et al. in 2010. In 2017, this instrument was translated into Chinese, and the Chinese adaptation showed acceptable reliability and validity. Comprising 15 items divided among three categories, the Chinese - version TFI includes 8 physical items, 4 psychological items, and 3 social items. The overall score spans from 0 to 15. If an individual scores 5 or more, it suggests the existence of frailty. Conversely, a score below 5 indicates the non - presence of frailty (9). To clarify the incidence of new−onset postoperative frailty, only patients without preoperative frailty (TFI < 5) were enrolled in this study. All patients underwent two assessments within 1 week preoperatively and on postoperative day 30 (or before the first cycle of adjuvant chemotherapy). The primary endpoint of interest was postoperative frailty, defined as the transition from no preoperative frailty (TFI < 5) to postoperative frailty (TFI ≥ 5). Based on this definition, patients were stratified into a postoperative frailty group and a non−postoperative frailty group.

### General information questionnaire

A detailed general - knowledge survey was crafted. It included the subsequent variables: the respondent’s age; the body mass index (BMI); and smoking history (Smoking history was defined as either having smoked more than one cigarette daily for longer than a year or having quit smoking within the past year) (answered as yes or no); alcohol consumption history (characterized by intake exceeding one standard drinking unit per day for more than one year or abstinence from alcohol for less than one year, with one standard drinking unit specified as 45 mL Baijiu, 360 mL beer, or 120 mL fruit wine) (yes or no); educational achievement divided into two groups: those with a high - school education or less, and those with a college education or more; marital status is grouped as either married or in other relationship statuses; monthly household income dichotomized as ≤5000 yuan or >5000 yuan; place of residence distinguished between urban and rural settings; medical payment method, either medical insurance or self-payment; and hypertension status, determined according to the diagnostic standards outlined in the Guidelines for Home Blood Pressure Self - monitoring (Second Edition) issued by the Japanese Society of Hypertension (10); yes ro no); hyperlipidemia (meeting the relevant diagnostic criteria in “Report of the Japan Atherosclerosis Society (JAS) Guideline for Diagnosis and Treatment of Hyperlipidemia in Japanese Adults” (11); yes/no); diabetes mellitus (meeting the relevant criteria in “Application of the Chinese Expert Consensus on Diabetes Classification in Clinical Practice” (12); yes/no); regular exercise (yes/no); clinical stage (Stage III/Stage IVB); pathological type (serous/other); pathological differentiation (poorly differentiated/moderately to well differentiated); presence of ascites (yes/no); and tumor diameter, treatment modality (primary cytoreductive surgery/interval cytoreductive surgery following neoadjuvant chemotherapy), completeness of surgical cytoreduction (R0/R1/R2).

### Nutritional status

Assessment was performed using the Mini nutritional assessment-short form (MNA-SF), a simplified version of the Mini nutritional assessment (MNA) scale. The Chinese version of the MNA-SF has a Cronbach’s α coefficient of 0. 84, demonstrating good reliability and validity. The scale consists of 6 items, including body mass index (BMI), recent eating status, and psychosocial issues, with a total score of 14 points. A score ≤ 7 indicates malnutrition, 8–11 points indicate nutritional risk, and a score ≥ 12 indicates normal nutritional status.

### Anxiety

The assessment was conducted using the state-trait anxiety inventory (STAI) (13), a tool comprising 40 items divided into two separate subscales: state anxiety and trait anxiety. Items 1 to 20 evaluate anxiety related to specific, immediate, or recent experiences, while items 21 to 40 measure more persistent emotional conditions. Answers are documented using a four - level Likert scale. The maximum possible total score on this scale is 160. As the scores increase, it indicates a greater intensity of anxiety symptoms.

### Sleep quality

Assessment was performed using PSQI (14). Conceived by Buysse and colleagues in 1989, this scale is crafted to assess the sleep conditions of patients and shows strong reliability and validity. It has an overall score that spans from 0 to 21, with a threshold of 7 points. As the scores increase, it indicates a decline in sleep quality.

### Detection of NETosis index

Key markers of NETosis were established as a comprehensive evaluation metric in this study. This index integrates three key markers involved in the NETosis process, including NE, MPO, and Cit-H3. All indicators are regarded as continuous variables, and higher values correspond to more active preoperative NETosis. Comprehensive assessment of these indicators enables a more thorough analysis of the association between systemic NETosis activity and the risk of postoperative frailty in patients with advanced ovarian cancer.

To optimally reflect the baseline status of patients and reduce interference from acute surgical stress, 5 mL of peripheral venous blood was collected from all patients in the early morning on the day before scheduled surgery under fasting conditions. Blood samples were placed in EDTA anticoagulant tubes and centrifuged at 3000 r/min for 10 min. Serum was isolated and stored at −80 °C until analysis. Concentrations of NE, MPO, Cit-H3, tissue factor (TF), von Willebrand factor (vWF), and the presence of P - selectin were ascertained via the enzyme-linked immunosorbent assay (ELISA). All procedures were performed in strict accordance with the kit instructions. Standard curves, blank wells, and duplicate wells were prepared for all samples. Absorbance values were read using a microplate reader, and the mean concentration was calculated for each sample. No single “NETosis index” was constructed in this study. Instead, three independent key biomarkers of NETosis were analyzed separately to more accurately reflect the activity of different stages in the NETosis process.

### Postoperative follow-up

Patients underwent follow-up evaluations at three-month intervals for a duration of two years subsequent to the completion of chemotherapy. The outpatient follow-up protocol encompassed assessments of tumor recurrence, alterations in tumor marker levels, and survival status. Additionally, telephone follow-up was employed to verify the most current patient health status.

### Statistical analysis

Statistical assessments and data visualizations were carried out utilizing SPSS 26. 0 and R 4. 3. 2 software. For continuous data that adhered to a normal distribution, an independent samples t - test was employed for analysis, and the results were presented in the format of mean ± standard deviation. The Mann -Whitney U test was employed to analyze continuous data that did not follow a normal distribution. The results were presented in the format of [*M(P25, P75)*]. Categorical variables were analyzed via the *x^2^* test and reported as frequencies(n), with a significance level set at *P* < 0. 05. *Kaplan-Meier* survival curves were constructed to evaluate the impact of postoperative frailty on prognostic survival. To address multicollinearity and preliminarily assess variable importance, *Pearson* correlation analysis was performed to examine the relationships between NETosis markers and nutritional status, anxiety, and sleep quality. Indicators with significant univariate differences were identified at *P* < 0. 05. Two machine - learning techniques, namely least absolute shrinkage and selection operator (*LASSO*) regression and extreme gradient boosting (*XGBoost*), were employed to screen differential indicators. Variables identified by both methods were selected for subsequent analysis, with the objective of obtaining a more robust set of candidate variables. These jointly screened variables were incorporated into a multivariate *Logistic* regression model. To mitigate the risk of overfitting, although feature selection was conducted via *LASSO* regression, the stability of the final model should be interpreted with caution due to the limitation of the number of positive events (n=41). A nomogram predictive model was developed based on the rms package. Given the limited number of events in this study (n = 41), internal validation was performed using the *Bootstrap* resampling method (1000 iterations) to ensure model stability with calculation of the optimism-corrected concordance index (C-index) and calibration slope. The goodness-of-fit, calibration, and clinical applicability of the model were assessed via the *Hosmer-Lemeshow* test, calibration curves, and decision curve analysis. All continuous predictive variables were entered into the model in their original linear form. The missing data rate in the study was less than 2%, and sequential mean imputation was used to impute missing data. Notably, all continuous predictive variables were incorporated into the model in their original linear form, and non-linear relationships were not explored, which constitutes one of the limitations of this study.

## Results

### Comparison of clinical characteristics between frail and non-frail groups

A comparative assessment of clinical metrics, such as BMI, was carried out between the cohorts of frail and non - frail individuals. The results of this assessment indicated that there were no differences that reached statistical significance. (P > 0. 05). Notable statistical disparities were detected in age, educational level, marital status, regular exercise, nutritional scores, STAI scores, PSQI scores, MPO, NE, and Cit-H3 between the two groups (P < 0. 05, **Table 1**).

**Table 1:** Comparison of clinical characteristics between frail and non-frail groups.

Variables		Frail group(n=41)	Non-frail group(n=148)	*x²/t/Z*	*P*
Age(years, *x̅ ± s*)		65. 35 ± 20. 45	54. 88 ± 15. 89	3. 501	0. 001
Body mass index(BMI, kg/m², *x̅ ± s*)		24. 29 ± 2. 50	24. 41 ± 2. 48	0. 274	0. 785
Smoking history(n)	Yes	5	10	1. 300	0. 254
	No	36	138		
Alcohol consumption history(n)	Yes	8	18	1. 462	0. 227
	No	33	130		
Educational level(n)	High school or below	27	60	8. 281	0. 004
	College or above	14	88		
High school or below(n)	Married	29	128	5. 666	0. 017
	Other	12	20		
Monthly household income(n)	≤5000 yuan	18	55	0. 615	0. 433
	>5000 yuan	23	93		
Place of residence(n)	Urban	22	89	0. 556	0. 456
	Rural	19	59		
Medical payment method(n)	Medical insurance	32	111	0. 162	0. 687
	Self-payment	9	37		
Hypertension(n)	Yes	6	11	2. 034	0. 154
	No	35	137		
Hyperlipidemia(n)	Yes	3	5	1. 227	0. 268
	No	38	143		
Diabetes mellitus(n)	Yes	5	8	2. 311	0. 128
	No	36	140		
Regular exercise(n)	Yes	15	83	4. 888	0. 027
	No	26	65		
Clinical stage (n)	Stage III	23	105	3. 238	0. 072
	Stage IVB	18	43		
Pathological type (n)	Serous	33	129	1. 168	0. 280
	Other	8	19		
Pathological differentiation (n)	Poorly differentiated	20	89	1. 696	0. 193
	Moderately/well differentiated	21	59		
Presence of ascites (n)	Yes	28	80	2. 658	0. 103
	No	13	68		
Tumor diameter(cm, *x̅ ± s*)		4. 15 ± 0. 45	4. 08 ± 0. 41	0. 947	0. 345
Nutritional score(points, *M(P25, P75)*)		6. 00(5. 00, 7. 00)	7. 00(6. 00, 9. 00)	3. 678	<0. 001
STAI scores (points *x̅ ± s*)		95. 85 ± 22. 65	83. 64 ± 20. 18	3. 337	0. 001
PSQI scores (points, *x̅ ± s*)		13. 59 ± 4. 35	10. 85 ± 3. 89	3. 888	<0. 001
vWF(ng·L^-1^, *x̅ ± s*)		476. 49 ± 60. 38	458. 73 ± 55. 89	1. 769	0. 078
P-selectin(pg·L^-1^, *x̅ ± s*)		461. 28 ± 77. 38	443. 69 ± 63. 89	1. 487	0. 139
TF(ng·L^-1^, *x̅ ± s*)		406. 34 ± 55. 32	389. 54 ± 52. 78	1. 785	0. 076
Preoperative Tilburg score (points)		4. 55 ± 0. 64	4. 38 ± 0. 50	1. 807	0. 072
NETosis index	MPO(pg·L^-1^, *x̅ ± s*)	415. 77 ± 62. 78	378. 39 ± 59. 95	3. 497	0. 001
	NE(ng·L^-1^, *x̅ ± s*)	372. 36 ± 56. 78	331. 17 ± 58. 14	4. 018	<0. 001
	Cit-H3(ng·L^-1^, *x̅ ± s*)	355. 85 ± 90. 16	290. 99 ± 67. 68	5. 029	<0. 001
Treatment modality (n)	Primary cytoreductive surgery	20	90	1. 910	0. 167
	Interval cytoreductive surgery following neoadjuvant chemotherapy	21	58		
Completeness of surgical cytoreduction (n)	R0	38	142	1. 174	0. 556
	R1	2	5		
	R2	1	1		

### Relationship between postoperative frailty and prognosis in patients with advanced ovarian cancer

**Figure 1:**
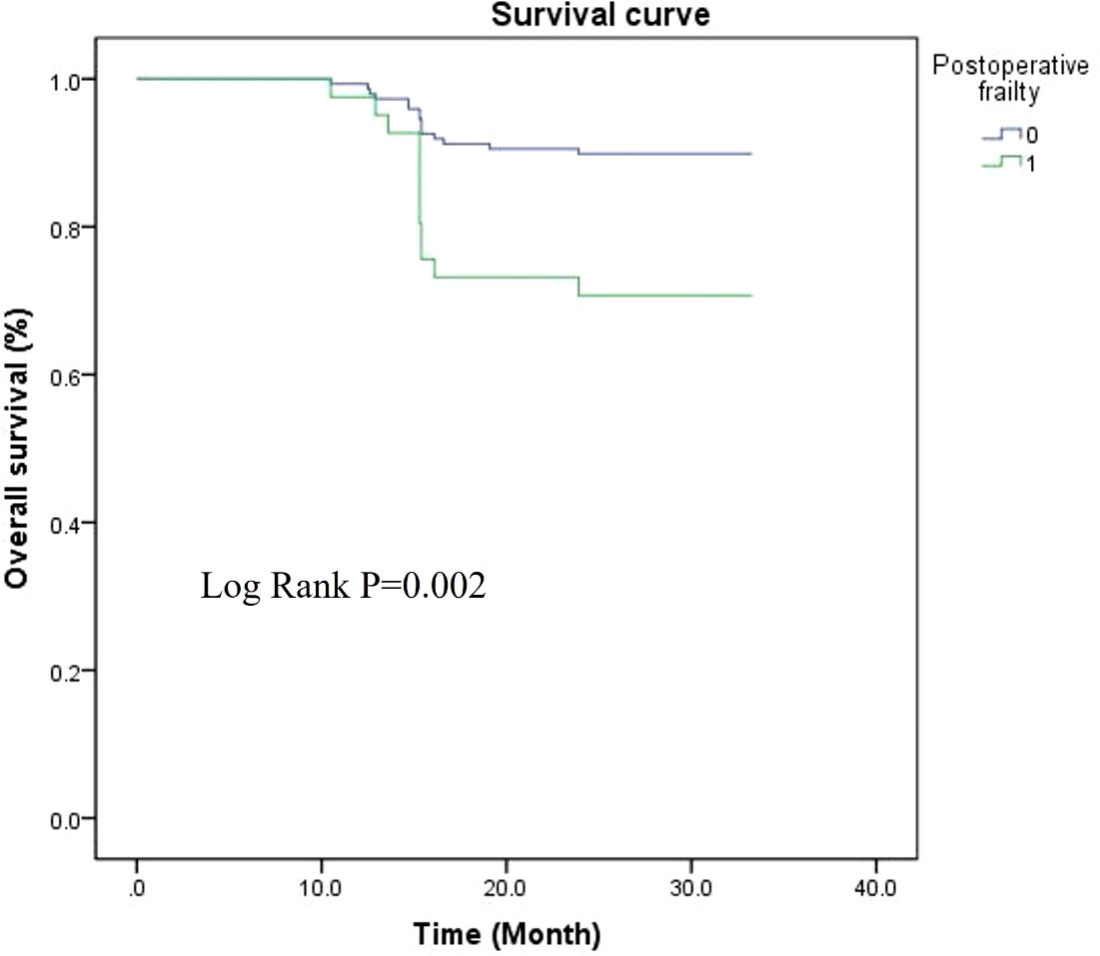
Association between post - operative vulnerability and prognosis among patients suffering from advanced ovarian cancer.

Based on exploratory analysis, the unadjusted Kaplan−Meier method was employed in this study to preliminarily assess the association between postoperative frailty and survival outcomes. The duration of the follow-up period for assessing the prognosis of patients with advanced ovarian cancer was two years. During this period, 27 patients (14. 29%) died before the end of follow-up, including 12 in frail group and 15 in non-frail group. Postoperative frailty was designated as the research factor, and survival status was defined by prognostic outcomes (0 = alive, 1 = dead) to assess the impact of postoperative frailty on survival time. Analysis of survival curves demonstrated that postoperative frailty was significantly correlated with adverse prognosis (Log-rank *P* = 0. 002 < 0. 05). This analysis was conducted as an exploratory study without adjustment for potential confounding factors including age and nutritional status. The results represent preliminary observational findings aimed at generating hypotheses for further research, and their clinical interpretation should be interpreted with caution. (**Figure 1**).

### Pearson correlation analysis

To exclude potential multicollinearity, the relationships between NETosis markers and nutritional status, anxiety, and sleep quality were examined using Pearson correlation analysis. No significant correlations were observed between MPO, NE, Cit-H3 and nutritional scores, STAI scores, or PSQI scores (all *P* > 0. 05), indicating no potential multicollinearity between NETosis markers and nutritional status, anxiety, or sleep quality, as shown in **Table 2**.

**Table 2:** *Pearson* correlation analysis.

Index		MPO	NE	Cit-H3
Nutritional score	Correlation coefficient	-0. 063	-0. 057	-0. 061
	*p*-value	0. 387	0. 435	0. 404
STAI score	Correlation coefficient	-0. 003	0. 133	0. 067
	*p*-value	0. 967	0. 068	0. 362
PSQI score	Correlation coefficient	0. 128	0. 060	0. 057
	*p*-value	0. 080	0. 410	0. 437

### Screening of important variables associated with postoperative frailty in advanced ovarian cancer via two machine learning algorithms

**Figures 2:**
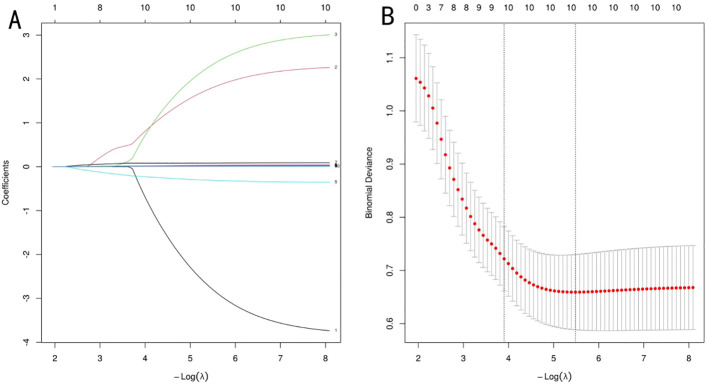
**(A, B)** LASSO regression analysis plots. Note: In **(A)** , a dual - axis layout along the horizontal dimension is utilized. The lower horizontal axis depicts the logarithmic scale of log(λ). Meanwhile, the upper horizontal axis showcases the equivalent number of variables that have been retained. The vertical axis displays the standardized coefficients for each feature, with their variation patterns depicted through colored curves for intuitive visualization. In **(B)** , the vertical axis denotes the mean squared error (MSE). The red vertical line marks lamb amin, signifying the optimal subset of variables associated with the minimum error. This particular point is equivalent to the quantity of independent variables incorporated into the model at the lowest MSE. The vertical black line, denoted as lambda. 1se, indicates the choice of a streamlined model when the error rises by one standard deviation.

**Figure 3:**
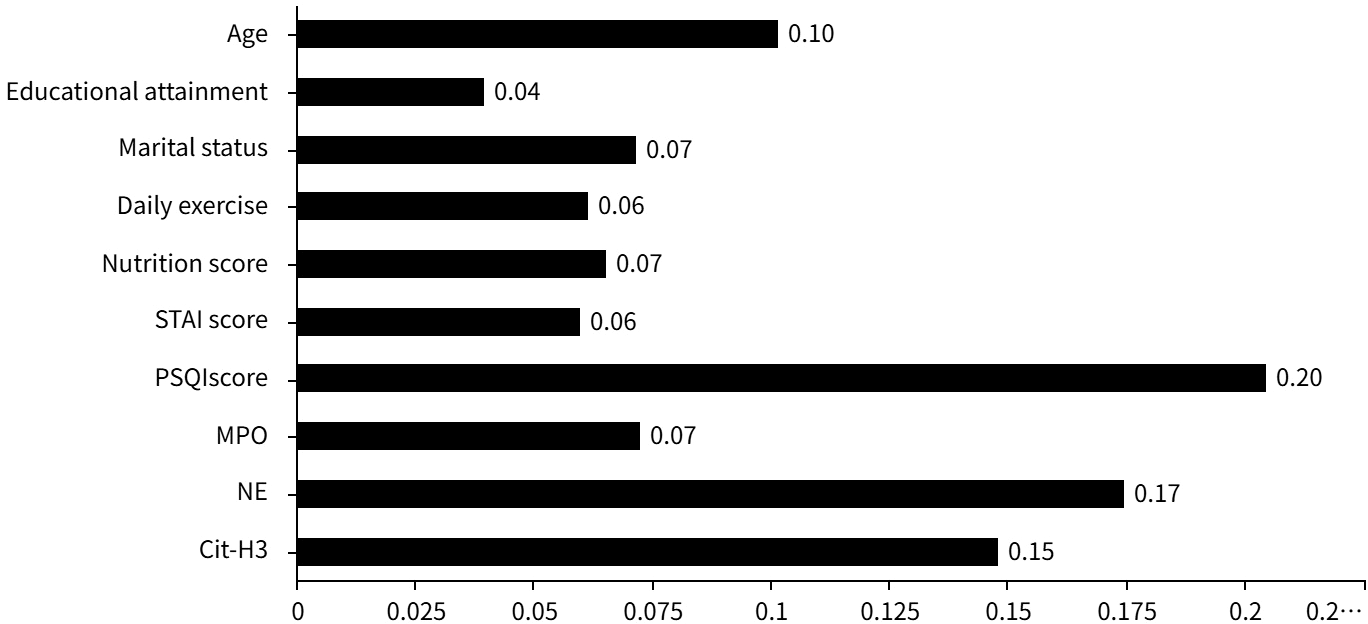
Plot of feature importance in XGBoost.

Based on the aforementioned data, postoperative frailty affects patients’ prognostic survival. Analysis of frailty-related factors facilitates delaying disease progression, reducing the risk of adverse events, and improving clinical prognosis through targeted interventions. To enhance model performance and mitigate multicollinearity, LASSO regression was employed for feature selection of initially screened differential variables, with regression coefficients of redundant variables shrunk to 0. To verify the reliability of the results, cross-validation was used to determine the optimal λ (**Figures 2A, B**). Ultimately, the λ value corresponding to the minimum error in ten-fold cross-validation (λ=0. 004) was selected as the optimal fitting value, with 10 variables included: age, educational level, marital status, regular exercise, nutritional score, STAI score, PSQI score, MPO, NE, and Cit-H3. *XGBoost* analysis revealed that age, regular exercise, nutritional score, STAI score, PSQI score, MPO, NE, and Cit-H3 had a weight ≥5%, demonstrating clinical interpretability. In contrast, educational level and marital status had a weight <5%, with insufficient model recognition efficiency, and thus these two indicators were excluded. *XGBoost* ultimately included 10 important variables: age, educational level, regular exercise, nutritional score, STAI score, PSQI score, MPO, NE, Cit-H3, and NETs (**Figure 3**). Parallel evaluation of important variables by the two machine learning methods was subjected to “overlap coverage” analysis, leading to the joint identification of 8 meaningful factors: age, regular exercise, nutritional score, STAI score, PSQI score, MPO, NE, and Cit-H3.

### Logistic regression examination of factors influencing post - operative frailty in advanced ovarian cancer

Variables identified through two learning techniques, namely *LASSO* and *XGBoost*, were incorporated into a logistic regression analysis. The results demonstrated that age (*OR* = 1. 035), STAI score (*OR* = 1. 028), MPO (*OR* = 1. 012), NE (*OR* = 1. 015), and Cit-H3 (*OR* = 1. 012) were all risk factors for postoperative frailty in advanced ovarian cancer (P < 0. 05). The nutritional score (*OR* = 0. 687) was determined to be a protective factor(P < 0. 05). Regular exercise and PSQI score did not reach statistical significance in the multivariate model (P>0. 05). (**Table 3**).

**Table 3:** Logistic regression examination of factors affecting post - operative frailty in advanced ovarian carcinoma.

Variables	Regression coefficient	Standard error	Wald χ^2^	*P* value	OR	OR 95% CI
Age	0. 035	0. 014	5. 906	0. 015	1. 035	1. 007 ~ 1. 064
Regular exercise	1. 020	0. 520	3. 851	0. 050	2. 773	1. 001 ~ 7. 678
Nutritional score	-0. 375	0. 130	8. 384	0. 004	0. 687	0. 533 ~ 0. 886
STAI score	0. 028	0. 013	4. 794	0. 029	1. 028	1. 003 ~ 1. 054
PSQI score	0. 121	0. 063	3. 665	0. 056	1. 128	0. 997 ~ 1. 276
MPO	0. 012	0. 004	8. 548	0. 003	1. 012	1. 004 ~ 1. 019
NE	0. 015	0. 005	10. 044	0. 002	1. 015	1. 006 ~ 1. 024
Cit-H3	0. 012	0. 003	12. 991	<0. 001	1. 012	1. 005 ~ 1. 018

### Development of a risk prognostic nomogram model for post - operative frailty in advanced ovarian carcinoma

**Figure 4:**
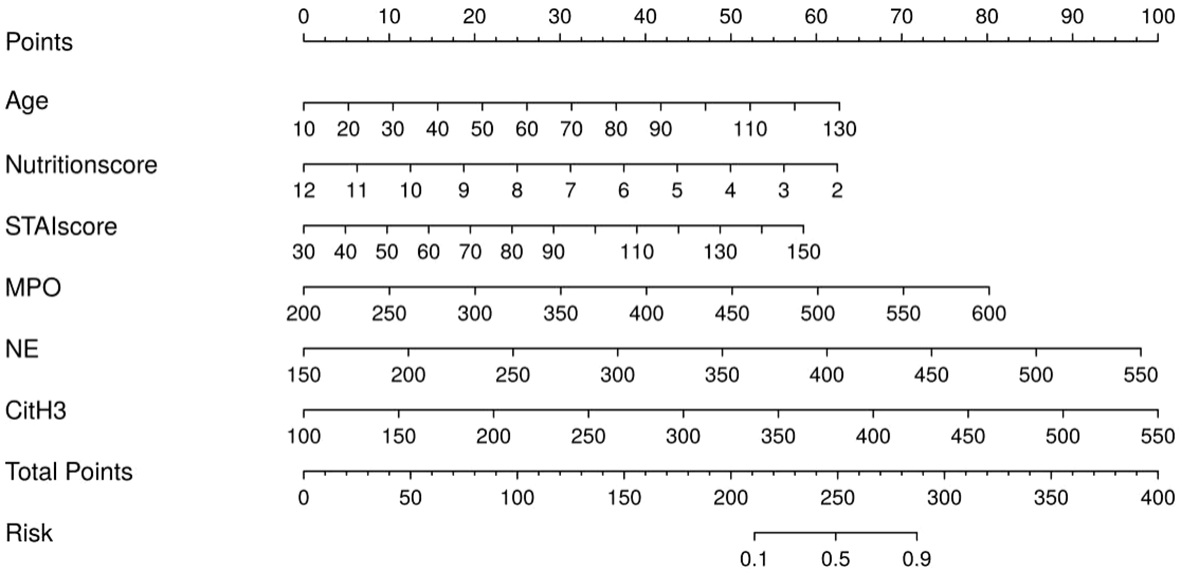
Risk prediction nomogram for postoperative frailty in advanced ovarian cancer.

Based on the aforementioned statistical analysis results, a nomogram model was established with the formula: In (p/1-*P*) = -18. 865-0. 375*nutritional score+0. 035* age+0. 028*STAI score + 0. 012*MPO+0. 015*NE+0. 012*Cit-H3. A visual scoring system was constructed in the study, with each risk factor corresponding to an independent scale axis. The length of the scale intuitively reflects the contribution weight of the factor to the occurrence of postoperative frailty, and the total score is obtained by summing the scores corresponding to each variable. The apparent AUC was 0. 882 (95%CI: 0. 826~0. 939), indicating a quantitative assessment that initially demonstrates favorable predictive efficacy for postoperative frailty risk in advanced ovarian cancer (**Figure 4**).

### Model performance evaluation

**Figure 5:**
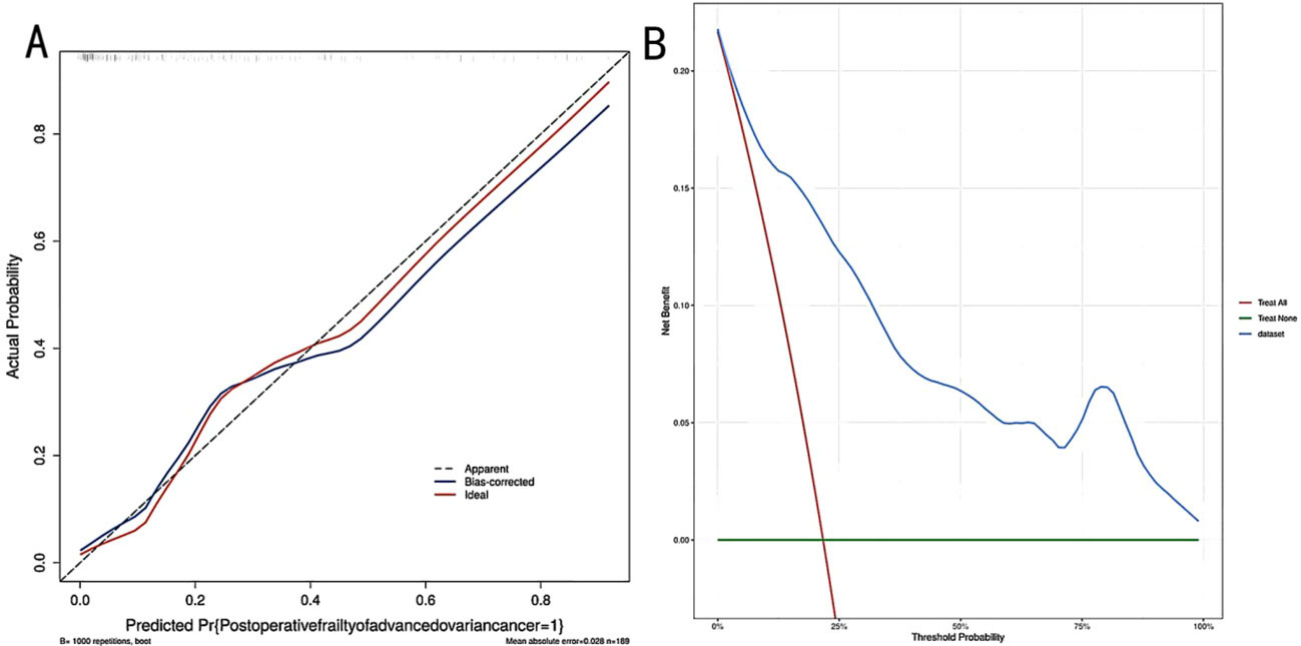
**(A, B)** Calibration curve and decision curve analysis (DCA) of the nomogram model.

The model’s performance was assessed through the *Bootstrap* technique, employing 1, 000 iterations of resampling. The optimism-corrected C-index of the model was 0. 856, and the calibration slope was 0. 92, indicating that the model still exhibited favorable discriminative ability and good calibration after optimism correction (the ideal calibration slope is 1). The *Hosmer-Lemeshow* test demonstrated goodness-of-fit of the model (*x²* = 3. 577, *P* = 0. 893). The calibration curve showed good consistency between predicted probabilities and actual observed probabilities (**Figure 5A**). Decision curve analysis revealed that the net benefit of the model was higher than that of the two extreme curves (“all patients postoperative frailty” or “no patients postoperative frailty”) across a wide range of threshold probabilities, which indicates that the model has a high net benefit. (**Figure 5B**).

## Discussion

Ovarian cancer represents one of the most prevalent malignant neoplasms affecting women. In China, approximately 52, 100 new cases are diagnosed each year, resulting in 22, 500 fatalities, thereby constituting the leading cause of mortality among gynecological cancers (15). Currently, optimal cytoreductive surgery serves as the core surgical approach for advanced-stage ovarian cancer. The maximal resection of all macroscopically identifiable tumor tissue facilitates the comprehensive removal of a substantial proportion of tumor cells within the body, thereby diminishing tumor burden and improving the effectiveness of chemotherapy, ultimately leading to increased patient survival rates (16). However, some patients are highly prone to frailty after surgery due to the influence of multiple factors. Wada H et al. (17) reported that frailty is regarded as a clinical syndrome independent of the normal aging process. Firstly, it heightens the likelihood of unfavorable consequences in elderly patients, such as suboptimal wound healing, accidental falls, bone fractures, and in severe cases, mortality. Secondly, it can trigger negative feelings like unease, pessimism, and social stigma, all of which impede the process of prognostic recovery. Conducting a comprehensive assessment of the elements that lead to postoperative frailty in patients diagnosed with advanced ovarian cancer is crucial. This assessment enables the early detection of high - risk groups and the formulation of customized interventions designed to improve clinical results.

NETosis refers to a reticular DNA structure decorated by granular proteins and histones that is released by activated neutrophils during the host immune response. Key biomarkers of NETosis include NE, MPO, and Cit-H3 (18). Consistent with the findings of Pan J et al. (19), elevated levels of NETosis-related key markers have been associated with tumor metastasis. In patients with advanced ovarian cancer, the tumor itself and therapeutic stress may create a chronic pro-NETotic environment. In the present study, data on key NETosis markers were analyzed. This analysis aimed to verify the predictive value of NETosis, a well-recognized pro-inflammatory and pro-catabolic process. The results demonstrated that preoperative levels of MPO, NE, and Cit-H3 were significantly higher in the frailty group than in the non−frailty group, and these markers were identified as independent risk factors for postoperative frailty. This observation can be explained by the fact that Cit-H3, MPO, and NE are core indicators involved in NETs formation or reflecting NETosis activity. These mediators promote thrombin generation and induce a hypercoagulable state, disrupt the normal tissue microenvironment through oxidative stress, and accelerate muscle protein catabolism, thereby directly leading to muscle atrophy and functional decline. Individuals diagnosed with advanced ovarian cancer frequently exhibit a chronic inflammatory state. Sustained presence of neutrophils by pro-inflammatory cytokines such as tumor necrosis factor (TNF) elicits systemic inflammatory responses and activates the sympathetic nervous system, resulting in local vascular endothelial injury as well as microthrombotic events. Cit-H3 promotes tumor cell invasion through epigenetic modifications, while the oxidative stress effects of MPO and NE disrupt the normal tissue microenvironment, accelerate the catabolism of muscle proteins, and directly induce muscle atrophy and functional decline (20). Furthermore, NETs can trap circulating tumor cells (CTCs) via their reticular DNA structure, release insulin-like growth factor-1 (IGF-1), increase intercellular adhesion, promote cancer cell aggregation, and stimulate dormant disseminated cancer cells. It also stimulates cancer cell proliferation through integrin signaling, activates relevant pathways such as NF-*κ*B, STAT3, and p38. trigger uncontrolled inflammatory responses, resulting in systemic inflammatory response syndrome (SIRS) and failure of multiple organs. thereby increasing the risk of postoperative frailty. In addition, factors associated with NETosis and the body’s hypercoagulable state include vWF, P-selectin, and TF. TF can initiate the extrinsic coagulation pathway. vWF binds to glycoprotein Ib (GPIb) on platelets, resulting in vascular wall adhesion and clot initiation. P - selectin triggers the release of NETosis - related key markers (NE, MPO and Cit-H3) by attaching to glycoprotein ligands present on the surface of neutrophils, and interacts with cancer cells, platelets, leukocytes, and endothelial cells to exert prothrombotic effect, which leads to the formation of a hypercoagulable state. This state exacerbates organ ischemia, reduces the attack of circulatory shear stress and natural killer (NK) cells on tumor cells, facilitates tumor cell metastasis and invasion, and increases the risk of postoperative frailty (21). In the current study, no statistically notable disparities were detected in the concentrations of vWF, P-selectin, or TF between the frail and non-frail cohorts (P > 0. 05). This discrepancy may be attributed to factors such as patient comorbidities, treatment cycles, disease stages, sample size, and follow-up duration in the current study, which differ from those in other clinical data. Additional studies are necessary to thoroughly assess the clinical relevance of these biomarkers within particular research settings. In the present study, a single composite index of NETosis was not adopted. Instead, the three key NETosis markers, MPO, NE and Cit-H3, were analyzed individually. The findings strongly suggest that activation of the NETosis process is not identical to ordinary granulocyte degranulation. Potential confounding factors were excluded during study design and statistical analysis. MPO, NE and Cit-H3 are endogenous substances present in patients before surgery, which can effectively reflect the activity of tumor status and chronic stress. Abnormal expression levels of NETosis markers indicate a higher risk of frailty in patients after complete cytoreductive surgery.

The data obtained in the current study indicated statistically significant differences in age, educational attainment, marital status, and engagement in regular physical exercise between the frail and non-frail cohorts (P < 0. 05). Prior research has demonstrated that older age is associated with poorer physical health and is frequently associated with multiple comorbid conditions. Abnormal secretion of angiogenic factors after surgery may simultaneously promote tumor angiogenesis and delay wound healing. Additionally, cognitive function, physical fitness, and physical activity decline to varying degrees with age. Elderly patients with advanced ovarian cancer exhibit reduced audio-visual function and manual dexterity, which impairs their self-care ability, increases psychological burden, and renders them more susceptible to physical and mental frailty (22). Furthermore, elderly patients have insufficient ovarian reserve, making follicular cells more vulnerable to the toxicity of chemotherapeutic drugs and accelerating the progression of premature ovarian insufficiency. In the meantime, the reduction in DNA repair ability associated with aging promotes the multiplication of remaining cancer cells. The trauma caused by surgery triggers an inflammatory reaction, which results in the release of pro - inflammatory substances. These substances then encourage the growth of the remaining cancer cells. The immune system function of patients decreases with age, weakening the ability to monitor cancer cells and increasing the risk of postoperative frailty. Patients with lower educational levels exhibited higher degrees of frailty. This may be attributed to limited access to information and knowledge regarding ovarian cancer and postoperative care among less educated patients, as well as poor self-care abilities. They cannot self-regulate effectively in the early postoperative period, lack awareness of utilizing social resources and health care, and thus fail to maintain their health adequately (23). Zhao B et al. (24) reported that marital status is correlated with the prognosis of ovarian cancer. Patients with unmarried, divorced, or widowed marital status may delay medical consultation due to lack of spousal or family support, resulting in higher tumor burden at diagnosis. During the postoperative recovery period, these patients without spousal care are highly prone to insufficient nutritional intake, poor compliance, and psychological depression. These factors exacerbate inflammatory responses and muscle catabolism, promoting the formation of a frailty phenotype. Furthermore, the absence of partner support among individuals who are unmarried, divorced, or widowed contributes to feelings of loneliness, which in turn activates the hypothalamic-pituitary-adrenal (HPA) axis, resulting in prolonged elevation of cortisol concentrations. This further suppresses immune function, accelerates muscle atrophy, and increases the risk of postoperative frailty. A significant association was identified between consistent physical activity and the occurrence of frailty, aligning with the results reported in numerous prior studies. Frail individuals exhibit physical function decline such as slow gait speed and gait instability (25). This observation may be explained by insufficient exercise accelerating muscle mass loss. Prolonged bed rest or limited activity after surgery inhibits muscle protein synthesis and enhances catabolism, worsening skeletal muscle atrophy. Meanwhile, a sedentary state exacerbates the continuous release of inflammatory factors, forming a chronic low-grade inflammatory environment. This further inhibits muscle regeneration, promotes ectopic deposition of adipose tissue, and leads to energy metabolism imbalance in patients, manifested as persistent fatigue and decreased exercise tolerance, thereby increasing the risk of postoperative frailty.

Multiple studies have identified STAI as a psychological assessment instrument. It is used to measure an individual’s transient emotional experiences in specific situations and relatively stable anxiety tendencies. The Pittsburgh Sleep Quality Index (PSQI) is for the purpose of conducting both clinical and fundamental research on the evaluation of sleep quality (26). The findings from the current study indicate that the scores on STAI and PSQI were elevated in the frail cohort compared to the non-frail cohort. The underlying mechanism involves complex interactions among psychological, physiological, and social factors. Anxiety itself is a manifestation of insufficient psychological and social coping resources and a component of psychological frailty, reflecting patients’ inadequate ability to cope with the stress of illness and surgery. This persistent psychological stress accelerates the progression of psychological frailty, thereby impairing overall recovery capacity. Previous studies have indicated that normal sleep-wake behavior promotes brain maturation and nervous system development. A correlation exists between sleep efficiency and frailty, with better sleep associated with a lower degree of frailty (27). Low sleep efficiency inhibits brain maturation and nervous system development, leading to impaired neural regulatory function. It directly disrupts endocrine balance, including the secretion of growth hormone (GH), insulin-like IGF-1, and testosterone reducing their secretion. This promotes muscle protein hydrolysis, accelerates muscle loss, decreases physical strength and physiological reserve, and renders the body more susceptible to the adverse effects of sleep disturbances, thereby increasing the risk of postoperative frailty. Relevant research has shown that anxiety and poor sleep quality form a vicious circle. Anxiety induces insomnia, while sleep deprivation further exacerbates anxiety, creating a self-reinforcing negative feedback loop (28). Patients during the postoperative rehabilitation period are inherently at risk of immunosuppression, metabolic disorders, and nutritional absorption disorders. Combined with the adverse effects of poor sleep and anxiety, these factors significantly impair their ability to cope with postoperative infections and delayed wound healing, thereby accelerating the progression of frailty. However, after analysis with the multiple-factor Logistic regression model in the present study, only the STAI score was retained as an independent influencing factor. This is attributed to the model’s adjustment for multicollinearity or confounding factors. A potential correlation exists between STAI scores and PSQI scores, but STAI scores more directly reflect the psychological mechanism of frailty in the model, leading to their priority retention. Additionally, data from the present study indicate that nutritional score is a protective factor against postoperative frailty in advanced ovarian cancer. Low nutritional scores suggest a higher risk of malnutrition in the body. Malnourished individuals experience further exacerbation of frailty due to inactivity, elevated cytokine activity, and muscle catabolism. Malnutrition not only accelerates physical frailty (such as fatigue and reduced walking speed) but also impairs eating function through oral frailty (including chewing dysfunction, dysphagia, and impaired olfaction and taste), forming a vicious circle of frailty and malnutrition (29).

Nomograms convert complex multivariate regression models into visual representations, enabling users to easily view and understand what were previously abstract data sets. This facilitates accurate prediction and supports clinicians in the decision-making process. Traditional nomogram models are constructed solely based on Logistic regression. While they can effectively identify high-risk groups, collinearity may exist between factors, leading to overfitting of the prediction model. In the present study, relevant factors were screened using two machine learning algorithms (*LASSO* and *XGBoost*), which eliminated linear relationships between indicators and effectively improved the scientificity and stability of the model. The calibration graphs indicated that the results predicted by the model were in close agreement with the data that was observed. Furthermore, the clinical decision curve consistently exceeded the two extreme reference curves, indicating a strong concordance between the model’s predictions and actual clinical observations, as well as a substantial net benefit. These findings provide robust evidence supporting the model’s utility in clinical decision-making. The data obtained in the current study, analyzed using Kaplan-Meier survival curves, demonstrated that the development of postoperative frailty is significantly correlated with an increased risk of adverse prognosis. This observation is presumably attributed to the fact that frailty is the result of the combined action of multiple factors and systems, among which chronic inflammation and epigenetics are important pathogenic mechanisms. Prior research has demonstrated that inflammatory responses are marked by increased concentrations of TNF, interleukins (ILs), and various other biomarkers within the organism. These responses affect the neuroendocrine system, promote catabolism, deplete amino acids in muscles, weaken stress responses and repair mechanisms, induce cachexia, and subsequently lead to functional decline of various organ systems (30). Moreover, epigenetic processes govern the distinct expression of genes within cells. This, in turn, initiates the appearance of phenotypes associated with aging. These phenotypes encompass the disruption of protein homeostasis, the malfunction of nutrient - sensing signaling pathways, mitochondrial impairment, depletion of stem cells, and modifications in intercellular communication. These changes result in physiological dysfunction and reduced viability, inhibiting postoperative functional recovery and increasing the risk of prognostic death. Furthermore, this study systematically collected baseline preoperative frailty data and included them as covariates in the analysis. The absence of significant differences in frailty index between the two groups before surgery indicates that the model identifies risk factors independently associated with the occurrence or significant exacerbation of postoperative frailty, rather than merely identifying individuals with preoperative frailty. This approach strengthens the explanatory power of perioperative biological factors such as NETosis in driving the transition to a frailty state. The established predictive model thus focuses more on identifying high-risk patients with a significant increase in postoperative frailty, providing more precise targets for targeted preoperative interventions.

In summary, age, nutritional score, STAI score, MPO, NE, and Cit-H3 were recognized as independent risk factors for postoperative frailty among patients suffering from advanced ovarian cancer. Postoperative frailty is closely associated with prognostic survival. The nomogram model constructed based on the aforementioned factors initially demonstrates favorable predictive efficacy and is expected to serve as an auxiliary tool for the early clinical identification of high-risk populations. Nevertheless, this study has certain limitations. This study adopted a small-sample size, single-center design, with a low number of positive events (n=41) and a low ratio of events to predictors (EPV≈6. 8). Despite LASSO selection, the risk of overfitting still exists. External validation remains absent, and the generalization ability of the model requires confirmation by multicenter studies. Linear assumptions were applied to continuous variables; variables such as age and STAI score were all incorporated into the model in a linear form, and potential non-linear relationships were not explored, which may affect model calibration. Residual confounding may exist: although no significant differences in treatment modalities and surgical completeness (R0/R1/R2) were observed in univariate analysis, these factors are theoretically associated with postoperative frailty. In addition, the survival analysis was exploratory and without multivariable adjustment, so the observed association between frailty and poor prognosis may be affected by multiple confounding factors. Future studies should address these limitations by adopting large-sample, multi-center study designs and external validation. Model development and validation should be conducted in larger multi-center cohorts in strict accordance with predictive model reporting guidelines such as TRIPOD.

## Data Availability

The original contributions presented in the study are included in the article/supplementary material. Further inquiries can be directed to the corresponding author.
